# Molecular Mechanisms Driving and Regulating the AAA+ ATPase VCP/p97, an Important Therapeutic Target for Treating Cancer, Neurological and Infectious Diseases

**DOI:** 10.3390/biom13050737

**Published:** 2023-04-24

**Authors:** Sepideh Valimehr, Ashish Sethi, Manjari Shukla, Sudipta Bhattacharyya, Mohsen Kazemi, Isabelle Rouiller

**Affiliations:** 1 Department of Biochemistry & Pharmacology, The University of Melbourne, Melbourne, VIC 3010, Australia; s.valimehr@unimelb.edu.au (S.V.);; 2 Bio21 Molecular Science and Biotechnology Institute, The University of Melbourne, Melbourne, VIC 3010, Australia; 3 Bio21 Ian Holmes Imaging Centre, Department of Biochemistry & Pharmacology, The University of Melbourne, Melbourne, VIC 3010, Australia; 4 ARC Centre for Cryo-Electron Microscopy of Membrane Proteins, The University of Melbourne, Melbourne, VIC 3010, Australia; 5 Australian Nuclear Science Technology Organisation, The Australian Synchrotron, 800 Blackburn Rd, Clayton, VIC 3168, Australia; 6 Department of Bioscience and Bioengineering, Indian Institute of Technology Jodhpur, Jodhpur 342030, Rajasthan, India

**Keywords:** p97-VCP, ATPase, cryo-electron microscopy

## Abstract

p97/VCP, a highly conserved type II ATPase associated with diverse cellular activities (AAA+ ATPase), is an important therapeutic target in the treatment of neurodegenerative diseases and cancer. p97 performs a variety of functions in the cell and facilitates virus replication. It is a mechanochemical enzyme that generates mechanical force from ATP-binding and hydrolysis to perform several functions, including unfolding of protein substrates. Several dozens of cofactors/adaptors interact with p97 and define the multifunctionality of p97. This review presents the current understanding of the molecular mechanism of p97 during the ATPase cycle and its regulation by cofactors and small-molecule inhibitors. We compare detailed structural information obtained in different nucleotide states in the presence and absence of substrates and inhibitors. We also review how pathogenic gain-of-function mutations modify the conformational changes of p97 during the ATPase cycle. Overall, the review highlights how the mechanistic knowledge of p97 helps in designing pathway-specific modulators and inhibitors.

## 1. Introduction

### 1.1. General Introduction

**Figure 1:**
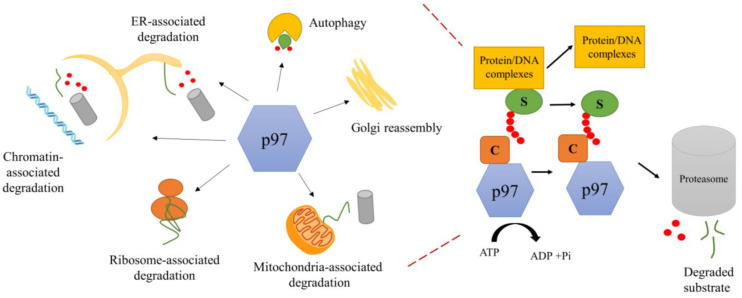
Diverse functions of p97 in the cell. p97 performs a variety of ubiquitin-dependent or -independent functions in the cell (ubiquitin schematically represented as a red circle) by using chemical energy from ATP hydrolysis to separate the substrate (S, green lines or oval shapes) from protein or DNA complexes and to prepare it for degradation through the proteasome. Numerous cofactors (C) assist p97 functions in substrate recruitment and processing.

The superfamily of AAA+ ATPases (ATPases associated with diverse cellular activities) regroups proteins with a common ~230-amino acid ATPase domain, the AAA domain [1]. Present in all organisms, AAA+ ATPases function by converting the chemical energy released from ATP hydrolysis into mechanical force, enabling these enzymes to perform a variety of cellular functions [2]. A prominent member of this family is the protein named p97 or VCP in mammalian and plant cells, with orthologs in all kingdoms of life, including *Mycobacterium smegmatis* (Msm0858, also known as MsmCpa), *Leishmania infantium* (LiVCP), *Trypanosoma brucei* (tbVCP), *Thermoplasma acidophilum* (VAT), *Saccharomyces cerevisiae* (Cdc48) and *Drosophila melanogaster* (TER94) [3,4,5,6,7,8,9]. p97 is one of the most abundant cytosolic proteins in all eukaryotic cells and has been shown to play a central role in gene expression regulation, organelle and protein biogenesis, and homeostasis. Examples of cellular pathways driven by p97 are shown in Figure 1. In recent years, the emergence of p97 as a target for treating cancer, neurodegenerative diseases and viral infections [9,10,11] has motivated studies to better understand the molecular mechanism by which p97 functions.

p97 and homologues undergo conformational changes in response to ATP binding and hydrolysis. Progress in understanding the mechanochemical cycle of p97 through biochemical and structural studies has started to reveal the molecular mechanisms regulating this cycle in health and disease conditions. Several dozens of cofactors/adaptors have been identified and are likely critical in defining the multifunctionality of p97 [12]. Some of these cofactors modulate the ATPase activity of p97, and the interaction of p97 with cofactors is modified when p97 contains disease-causing mutations [13]. Several inhibitors of p97 have been identified [14], and their mode of action is starting to emerge. The purpose of this review is to provide an overview of the molecular mechanisms driving and regulating p97’s activity and how the current inhibitors function. A better understanding of these mechanisms would allow for the design of more specific inhibitors and even pathway-specific inhibitors that only target particular functions of p97 and not others.

### 1.2. Biological Function of p97

p97 is involved in a large variety of cellular pathways (with examples shown in Figure 1). p97 was first identified as a crucial component for the homotypic fusion of membranes in cell division to maintain intact organelles [15,16]. It is required to assemble Golgi cisternae [17] and for the turnover of outer mitochondrial membrane proteins [18]. p97 extracts proteins from the chromatin and contributes to genome stability [19,20]. It performs a crucial role in the endoplasmic reticulum-associated degradation (ERAD) pathway [21]. p97 acts on a diverse range of substrates to keep the protein balance in the cell and performs a variety of functions by interacting with different cofactors, many of which are ubiquitin ligase or deubiquitinase [22,23]. Substrates include numerous polyubiquitinated misfolded proteins [24]. Some p97 cofactors have been shown to function as substrate-recruiting or substrate-processing cofactors [12]. p97 has also been shown to act upstream of the proteasome to prepare the substrate for degradation through the proteasome by providing a flexible initiation region [25]. For decades, researchers were intrigued by the ability of p97 to be involved in so many pathways until elegant in vitro studies of the yeast homologue, Cdc48, and human p97 demonstrated the ability of Cdc48/p97 to unfold ubiquitinated substrates [26,27]. These studies pointed to a common molecular activity of Cdc48 as an unfoldase, with the ability to unfold a multitude of substrates involved in many varied cellular pathways.

### 1.3. Structure of p97

**Figure 2:**
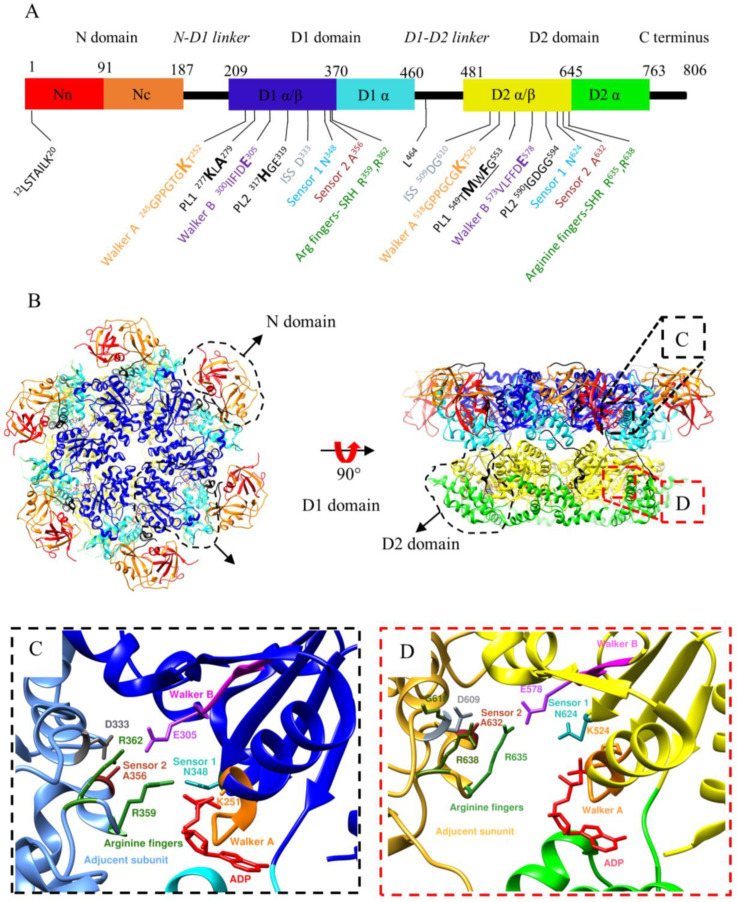
Domain organisation, hexameric structure and AAA module of p97. (**A**) The monomer consists of an N domain (with one Nn (red) and one Nc (orange) subdomain), an N-D1 linker (black), a D1 domain (with a D1α/β (blue) and D1α (cyan) subdomains), a D1-D2 linker (black) and a D2 domain (with one D2α/β (yellow) and one D2α (green) subdomain), and a disordered C-terminal region (black). The D1α/β and D2α/β subdomains contain the structural motifs involved in nucleotide binding (Walker A), hydrolysis (Walker B) and sensing (arginine fingers, ISS, sensor 1 and sensor 2) as well as pore loops (PL1 that interacts with substrates and PL2). Residues at the N-terminus rearrange from disordered to a short helix associated to stabilise the movement of the N domains. L^464^ in the D1D2 linker is a critical residue in coordinating the ATPase activity of D1 and D2. (**B**) The hexameric structure of p97 is shown from the top and side views using the same colour code (PDB ID: 5FTK). (**C**,**D**) Insets focus on the nucleotide binding and hydrolysis motifs in the D1 and D2 domains. The nucleotide has been removed for clarity. The Walker A and B motifs, arginine fingers, sensor 1 and sensor 2 loops are shown in orange, fuchsia, green, cyan and brown, respectively. Of note, the arginine fingers (also referred to as the SRH motif) and the sensor motif are located on an adjacent protomer to that containing the sensor 1 and the Walker A and B motifs.

Low-resolution cryo-EM studies reported almost 20 years ago that the quaternary structure of p97 is a homohexamer forming a double barrel composed of two stacked rings [28,29]. The first high-resolution structures of p97 were determined using X-ray crystallography at 4.7 Å in 2003 [30] and 2.3 Å in 2016 using single-particle cryo-EM [31]. These structures showed that the monomer consists of the N domain (residues 1–187), N-D1 linker (residues 188–208), D1 domain (residues 209–460), D1-D2 linker (residues 461–480), D2 domain (481–763) and a disordered C-terminal region (residues 764–806) (Figure 2A). They confirmed the overall stacked ring organisation of the quaternary structure p97 (Figure 2B), with one ring formed by oligomerisation of six D1 domains, and the other by oligomerisation of six D2 domains. These structures also defined the structural motifs of p97 (Figure 2C,D). Structural motifs of p97, characteristic of the AAA+ superfamily, include motifs responsible for nucleotide binding, nucleotide sensing and ATP hydrolysis. The consensus sequence of the Walker A motif is GxxGxGK [S/T], with G, K, S and T denoting glycine, lysine, serine and threonine, and x any residue. This motif is between the first strand of the core β-sheet and the following helix. The consensus sequence of the Walker B motif is ϕ4DE, with ϕ being any hydrophobic residue, D an aspartate and E a glutamate. The Walker B is associated with the third strand of the β-sheet. The arginine residues named arginine fingers are located in the proximity of the γ-phosphate of the bound ATP in the adjacent subunit and stabilise the transition state during the interaction with the adjacent subunit (Figure 2C,D). Sensor 1 (N^348^ and N^624^) and sensor 2 motifs (A^356^ and A^632^) play a role in nucleotide binding/hydrolysis through coordination with walker B [32,33,34]. Intersubunit signaling (ISS) motif (N^333^ in D1 and D^609^ in D2) coordinates ATP hydrolysis between protomers via a network hydrogen bonding with R^362^ and E^305^ in the D1 domain; G^610^, D^609^, E^578^ and R^635^, K^543^ in the D2 domain [33]. Of note, the nucleotides bind between two adjacent subunits with the arginine fingers, the ISS and sensor 2 motifs located in the protomer adjacent to that containing the Walker A, Walker B, and Sensor 1 motifs, indicating cooperativity between the AAA domains with each of the two stacked rings in p97.

For the AAA+ ATPase dynein that contains similar structural elements, the mechanism of ATP hydrolysis starts with the absorption of the proton from a water molecule by the glutamate residue of Walker B and then the water molecule will be activated for nucleophilic attack on the γ-phosphate of bound ATP; the conserved residues in Walker A bind to β- and γ-phosphates and Mg^2+^ ions. The binding and hydrolysis of ATP happen at the interface between domains, and this causes conformational changes leading to the protein function [35].

The D1 and D2 domains are connected by a linker, the D1-D2 linker. The flexibility of this linker is critical to signal transmission between the two ATPase rings [36], as described in detail below.

In the absence of a substrate, the central cavity of p97 is lined by four pore loops that form four sites of restrictions in p97’s central cavity. There are two pore loops per AAA domain: pore loop 1 (PL1) and pore loop 2 (PL2). The PL1 of AAA+ ATPases is conserved and contains a ΦXG signature motif (where Φ is an aromatic amino acid, X is any amino acid and G is a glycine). However, in p97 and homologues, this motif is found only in the PL1 of the D2 (PL1-D2) domain ^549^WFG^553^ and not in the PL1 of the D1 domain (PL1-D1). The pore formed by PL2 loops is narrower than that formed by PL1 loops. H^317^ lines the narrowest restriction of the D1 ring. In addition, PL2 residues, H^317^ (D1), R^586^ and R^599^ (D2), are essential in the ERAD pathway [37].

Residues of the PL1-D1 and PL1-D2 of p97 (and Cdc48) have been shown to interact with the substrate unfolded polypeptide chain, despite the absence of a conserved motif in PL1-D1 [38,39,40]. The structures of p97 and Cdc48 in complex with an unfolded substrate are described in detail below.

## 2. The Mechanochemical Cycle of p97

**Figure 3:**
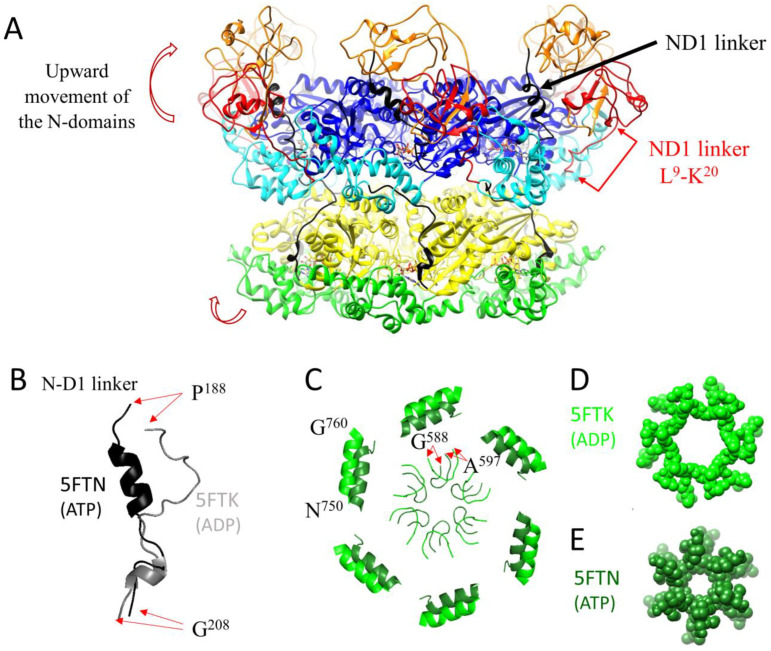
Conformational changes of p97 during the ATPase cycle in the absence of a substrate. (**A**) Conformation of p97 with ATP-bound D1. Compared to Figure 2 (PDB ID: 5FTK/ADP), ATP binding (PDB ID: 5FTN/ATP) to D1 induces an upward movement of the N domain upon (**A**), a change of conformation of the N-D1 linker (P^188^–G^208^) (**B**), inward displacement of α9 (N^750^–Q^760^) and closing of the D2 pore formed by loop G^588^–A^597^ (**C**), with residues of the loops shown as spheres in (**D**,**E**).

**Figure 4:**
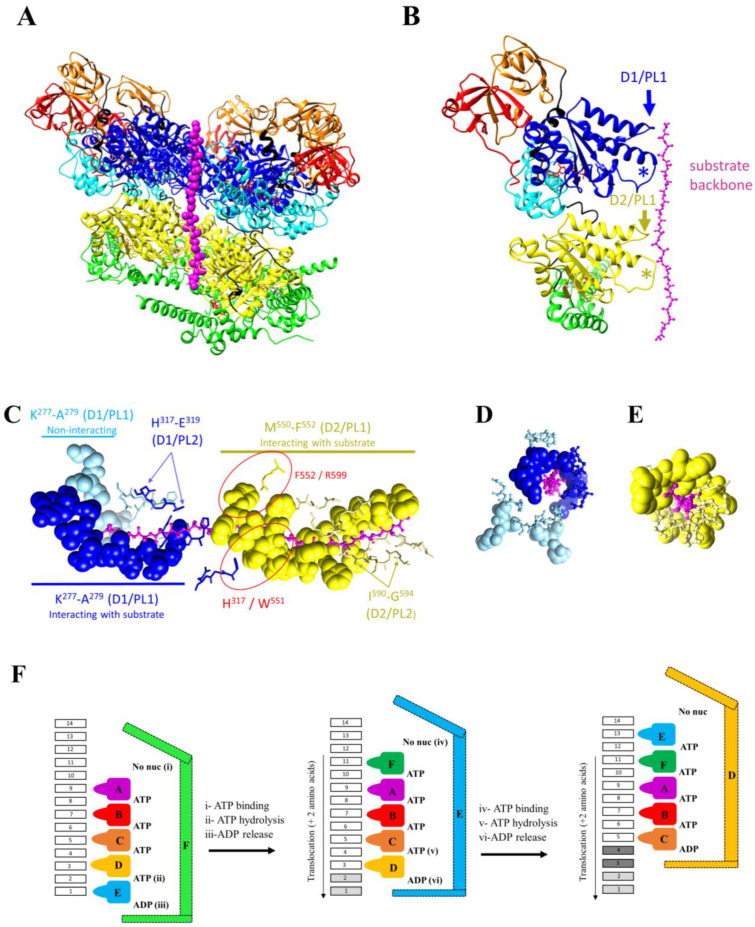
Structural changes of p97 upon substrate binding. (**A**) Structure of the human p97 interacting with an unfolded substrate (PDB ID: 7LN6). Compared to Figure 3A, five of six subunits rearranged from a rotational to helical symmetry upon substrate binding. The sixth subunit, which exists in a range of positions, has been removed for clarity. The substrate was observed in an extended β-strand conformation and is shown as magenta beads with its long axis vertical in the plane of the screen. (**B**–**E**) Pore loops and residues interacting with the unfolded substrate. The unfolded substrate is shown in the plane of the page (**C**) and perpendicular to the plane of the page (with (**D**,**E**) showing pore residues in the D1 and D2 ring respectively). Residues in the pore loop 1 (PL1) of D1 (K^277^–A^289^, shown as blue spheres, in (**C**) with the unfolded substrate in the plane of the page and in (**D**) with the unfolded substrate perpendicular to the plane of the page) and D2 (M^550^–F^552^, shown as yellow spheres (**C**,**E**) with the unfolded substrate in the plane of the page) interact via hydrogen bonds with the translocating substrate backbone. Pore loop 2, PL2 (**B**), residues ^317^HGE^319^ for D1 and ^590^IGDGG^594^ for D2, shown as sticks (**C**–**E**), form an ~16 Å diameter channel around the substrate with a groove on one side. Two H^317^ in D1-PL2 also form hydrogen bonds with the translocating peptide (dark blue). π–π stacking between H^317^ of D1/PL2 and W^551^ of D2/PL1 may help in coordinating D1 and D2 unfolding activity. π-stacking of R^599^ (close to D2-PL2) with F^552^ in D2-PL1 may help in coordinating the two loops in D2. (**F**) Orthodox model for substrate translocation derived from the yeast structure (PDB ID: 6OPC). Five subunits (**A**–**E**) interact with the substrate every two amino acids (1–10) via the PL1 loop.

Recent technological advances in the field of cryo-EM have allowed us to further our understanding of the mechanochemical cycle of p97 by determining over 50 structures of full-length p97/Cdc48/VAT in different conformations along the ATPase cycle [31] and bound to substrates and cofactors [38,39,40]. Below, we describe in detail these conformational changes. Overall, in the absence of a substrate, conformational changes observed include upward and downward movement of the N domain, closing and opening of the D2 pore, and rotation of the D1 and D2 rings with respect to each other (Figure 3). Upon interaction with a substrate, the general organisation of p97 changes from a two-stacked ring assembly into a helical staircase/split-washer conformation (Figure 4).

### 2.1. Conformational Changes during the ATPase Cycle of p97

ATP binding and the associated conformational changes are allosterically and cooperatively coordinated. Initial mutagenesis studies of the Walker A and Walker B motifs suggested that the D2 domain of p97 had the major ATPase activity and that D1 did not hydrolyse ATP [41]. Further studies revised this concept and demonstrated that nucleotide binding to D1 enhances the ATPase activity of D2, whereas ATP binding or hydrolysis by D2 enhances the D1 ATPase activity by promoting the release of a tightly bound ADP [42]. D1 has a high affinity for ADP (~1 × 10^−6^ M), whereas D2 has a lower affinity for ADP (90 × 10^−6^ M) and can readily exchange ADP for ATP. The D1-D2 linker transduces the response to ATP binding in D2 to D1 via interprotomer communication [43], favouring ADP release by D1 and subsequent ATP binding [44]. The current literature supports that ATP binding to D1 triggers the upward movement of the N-domain from its coplanar state (Figure 3A) and ATP binding to D2 constriction of the D2 pore (Figure 3B) [31,33,45,46]. Concurrently with the upwards movement of the N-domains, the structure of the N-D1 linker changes from a loop to a three-turn α-helix (R^191^-D^200^) (Figure 3B) [45]. The disordered N-terminal peptide L^9^-K^20^ of the N-domain in the ADP-bound state reaches the hydrophilic tunnel between the first helix of the related D1-domain (α4) and the helix (α12) from a neighbouring D1-domain (Figure 3A).

Upon ATP binding to the D2-domain, the size of the restriction formed by PL2 of D2 changes from 16 to 8 Å with an inward displacement of helix 750–760 [31] (Figure 3C). Removal of nucleotides to all 12 nucleotide binding sites leads to p97 forming a dimer of hexamers, with the N-domains adopting an upward conformation, as shown by NMR spectroscopy and single-particle cryo-EM [47,48,49]. Gao and co-authors [49] proposed that the dodecamer could be a potentiated state ready for substrate engagement. Yu and co-authors [48] speculated that dodecamerisation might be a mechanism that favours activities of p97 in which only segregation of a substrate is required, such as membrane fusion, over activities that require substrate unfolding or threading through the pore. Yu and colleagues also proposed that the dodecamer could be a storage form of the protein. The biological relevance of the dodecamer and nucleotide-free conformation remains unknown, and further studies are required to elucidate it.

### 2.2. Conformational Changes of p97 upon Substrate Binding

All the structures of p97 solved without substrates showed D1 and D2 as concentric rings, whether the structures were hexamers or dodecamers (Figure 2 and Figure 3). However, the overall organisation of p97 is dramatically different after interaction with a substrate: the six-fold symmetry is broken, and the subunits are arranged with respect to each other following a semi-helical pattern.

Cryo-EM studies conducted with the yeast Cdc48 [38,39] and the human p97 [40,50] homologues showed a single unfolded polypeptide chain in the central cavity of the complex. These conformations were captured with native substrates and cofactor Shp1 with Cdc48 and cofactor p47 with human p97 [38,50] or artificial ubiquitinated substrates and cofactors Npl4-Ufd1 [39,40]. Over 25 structures of p97 with bound-engaged substrates have been reported. As reviewed by Gates and Martin [51], residues of PL1 and PL2 are conserved among different AAA+ ATPases. Typically, residues of PL1 interact with the substrates, with five out of the six PL1s of the ring formed by six AAA domains interacting with the substrate, forming a staircase around the unfolded substrate. The rise between PL1 from adjacent monomers is ~6Å, a distance favouring the interaction of the loop every two amino acids along the unfolded substrate chain. PL2 residues arrange in a staircase similar to PL1 but do not directly interact with the substrate.

Similar to other AAA+ ATPases, upon interacting with a substrate, p97 monomers rearranged from a six-fold rotational symmetry (C6) into a semi-helical symmetry arrangement (Figure 4A) with four to six D1 and D2 domains packed into the helical pattern. The monomers interact with the substrate via residues in PL1 (M^288^–A^289^ and W^561^–Y^562^ in yeast D1 and D2, respectively; K^277^–L^278^–A^279^ and M^550^ and F^552^ in human D1 and D2, respectively) (Figure 4B–E). Despite differing from the consensus sequence, residues of PL1-D1 of Cdc48 and p97 also interacted with the substrate backbone (Figure 4B–E).

In yeast Cdc48, five PL1-D1s and PL1-D2s interacted in a staggered fashion with the substrate. The sixth PL1 belongs to an AAA domain poorly defined on the EM map [38]. Using cryo-EM classification methods, Cooney and co-authors showed that this domain adopted two preferred positions, both located between the upper and lower AAA domains interacting with the substrate along the length of the helix, and with both positions off-centred so that the PL1 of this domain did not interact with the substrate.

In these structures, ATP is located between the five AAA domains that interact with the substrate via the PL1 loop. On one side of this block of five domains, there is no nucleotide, while ADP is located on the other side. The sixth AAA domain does not interact with the substrate and is flexible. This domain adopts several transitional positions, with two of these being more highly populated than the others [38]. This conformation suggested the hand-over-hand model of translocation [38], where successive ATP binding, hydrolysis and ADP release would result in the translocation of two amino acids through p97 pores (Figure 4F). For simplicity, Figure 4 shows the reactions occurring in one of the two AAA domain rings. The Cdc48 structure obtained with the nucleotide analog ADP-BeFx suggests that the D1 and D2 rings undergo similar conformational changes in the cycle, with the hydrolysis of one molecule of ATP and the release of one molecule of ADP per cycle per ring. This being so, the translocation of two amino acids through the Cdcd48 central pore would be accompanied by the hydrolysis of two ATP molecules and the release of two ADP molecules. It is worth noting that this model is derived from a single structure obtained in the presence of a nucleotide analog, which could have captured a dead-end conformation. Further studies, such as biophysical approaches that combine optical tweezers with single-molecule fluorescence detection, are required to correlate the translocation rate with the number of ATP molecules hydrolysed.

Structures obtained with p97 suggest that subunits of the D1 and D2 rings may not move simultaneously [40]. In these structures, only four D1 domains interacted with the substrate. The D2 ring was resolved in two conformations, called the open and closed conformations by the authors. In the open conformation, all six subunits interacted sequentially with the substrate, and the D2 ring was broken. In the closed conformation, five subunits interacted with the substrate. The sixth subunit was off-centred and connected with the two subunits at the extremities of the staggered subunit helix. Residues of PL2 lined the pore but did not interact with the substrate, except for H^317^ in two PL2s. Mutagenesis studies showed that residues in PL2-D2 were not essential for p97 unfoldase activity [40]. However, residues in PL2 may coordinate unfolding by the D1 and D2 rings. In particular, H^317^ (PL2-D1) formed a π–π interaction with W^551^ (PL1-D2) and F^552^ (PL1-D2) was π-stacked with R^599^ (Figure 4C), and these interactions were shown by mutagenesis to be essential for unfolding [40]. The overall mechanism of unfolding (Figure 4F) is supported by the structures of human p97, as ATP is always found between staggered domains that interact with the substrate. In the two human conformations, ATP/ADP was found between subunits that did not interact with the substrate. These differences may be due to affinity differences, but this agrees with the hand-over-hand unfolding model. It is to be noted that different conformations (nine) were obtained in the presence of different detergents and with two mutations in p97 (A232E and E578Q) to stabilise the complex. Further biophysical studies are required to place these structures in the cycle of protein unfolding. Nonetheless, the presence of different conformations suggests a certain degree of malleability and adaptability in p97 to allow the processing of a large range of various substrates, including large, branched, polyubiquitinated proteins. Moreover, in these structures, novel interactions necessary for the unfoldase activity were observed, notably between the N and C-terminal tails of p97 and the adjacent p97 subunit, namely and respectively, with an acidic patch in the α-subdomain of the D1 domain and a groove in the α-subdomain of the D2 domain.

### 2.3. The Orthodox Model of Substrate Unfolding

A model has been proposed to explain substrate unfolding by a single AAA ring. According to this model, referred to as the orthodox model of substrate unfolding (Figure 4F), five subunits (A–E) interact with the substrate every two amino acids via the PL1 loop. Four ATP molecules are located between these five subunits. The sixth subunit (F) is flexible and preferably adopts two positions, the lower and upper positions, and presumably switches between these two positions. ADP is located between subunits E and F. No nucleotide is found between subunits F and A. According to this model, ATP binding between subunit A and F (i) would stabilise the F subunit in the upper position where it is able to interact with the amino acid (11) positioned two amino acids upstream of that interacting with subunit A (Figure 4F, middle panel). Concurrently or sequentially, ATP hydrolysis (ii) between subunits D and E and (iii) ADP release between subunits E and F would destabilise subunit E. This subunit (E) is freed up, becomes mobile and no longer interacts with the translocating polypeptide change. Its position fluctuates along the length of the helix between the lower position (now occupied by subunit E) and the upper position (now occupied by subunit F). The ATP binding cycle, hydrolysis and ADP release would repeat, allowing for the translocation of two amino acids per cycle (right panel). As this model is derived from a single structure, the order in which ATP, ATP hydrolysis and ADP release occurs is currently not known.

In this model, and for simplification purposes, only one of two AAA rings is described. The structures available for Cdc48 suggest that the two rings function in a similar and coordinated manner. However, while supporting this model, the structures obtained with human p97 suggest that the unfoldase activity of the D1 and D2 rings is coordinated but can occur asynchronously in the two ATPase rings [40]. Since the interaction of residues in PL1-D2 with the substrate is more extensive than that of PL1-D1, the D2 ring has been proposed to be the main driver of the unfoldase activity, with mutations in PL1-D1 reducing but not abolishing p97 unfoldase activity.

## 3. Regulation of p97 Activity

### 3.1. Regulation of p97 Activity by Cofactors

To perform different functions in the cell and process different substrates, p97 interacts with a variety of cofactors. A large number of cofactors (>40) have been shown to interact with p97. Previous reviews have described the protein domains involved in the interaction of cofactors with p97, their presence in different cofactors and their binding properties to the N and/or C-terminal domains of p97 [52,53,54]. Cofactor domains interacting with p97 include the UBX (ubiquitin regulatory X)/UBXL (UBX-like), PUB (Peptide:N-glycanase/UBA or UBX-containing protein) and PUL (PLAP, Ufd3p and Lub1P) domains and the SHP box (also known as BS1, binding site 1) and the VIM (VCP-interacting motif) and VBM (VCP-binding motif) motifs [52].

The general principles driving cofactors’ interaction with p97 have started to emerge. Some cofactors have been reported to bind in a mutually exclusive fashion, while others bind cooperatively. For example, the cofactors p47 and Ufd1-Npl4 both interact via their UBX domain to the N-terminal domain of p97 in a mutually exclusive fashion [55]. Hierarchical binding, where one cofactor promotes the binding of a second cofactor, has also been reported; for example, the UBX cofactor FAF1 and UBDX7 only bind to p97 bound to cofactors Ufd1/Npl4, even though interaction occurs via the UBX of these three cofactors [20,53]. The stoichiometry of cofactor binding also varies, ranging from one heterodimer of Ufd1/Npl4 [39] to up to six p47 cofactors per p97 hexamer [28,47,56,57]. Although all of the 13 mammalian UBX (ubiquitin regulatory X) domain-containing proteins have been shown to interact with p97 [23], not all p97 cofactors contain a UBX domain and not all UBX containing cofactors interact with p97 via their UBX domain. For example, the cofactor UBXD1, which contains a UBX domain at its C-terminus, does not interact with p97 via this domain. Instead, UBXD1 has also been reported to bind to the C-terminus and N-terminal domain of p97 via its PUB domain [58] and via a canonical VIM motif and two N-terminal helices, respectively [59]. A recent study also revealed that the cofactor p47 also interacts with two adjacent p97 monomers using different motifs/domains, namely, a UBX domain and two SHP motifs, SHPN and SHPC [47]. A cryo-EM study of human p97 implies that Npl4 interacts with p97 through its zinc finger motifs and undergoes conformational changes similar to a seesaw motion, which are important for substrate unfolding by p97 [60].

The exact molecular mechanism of cofactor interaction with p97 in a specific pathway has been the subject of several reviews [12,61]. Accumulating evidence points to cofactors being important for pathway specificity, but further investigation is still required to gain a complete understanding of the selection process. Certain cofactors are essential for specific biological functions and dispensable for other functions. However, what controls specificity remains unclear. Some potential reasons for specificity have been evoked, such as the selection of mono- versus polyubiquitinated substrates, activation of the ATPase activity by cofactors, and cooperativity between nucleotide occupancy and cofactor binding, for example, for the cofactors p47 and Ufd1-Npl4 that mediate Golgi membrane fusion and endoplasmic reticulum-associated degradation in interaction with p97, respectively [57,62]. In contrast to p47, Ufd1-Npl4 activities depend on the proteasome. p47 recognises monoubiquitinated substrates, while Ufd1-Npl4 recognises polyubiquitinated substrates [63]. p37 recruits substrates to p97 which are not ubiquitinated [64].

Regulation of the p97 ATPase activity by cofactors is suggested by observations that showed that the p47 cofactor inhibits p97 ATPase activity [49], with higher inhibition at low rather than high concentrations of p47 [12], and that the p37 cofactor activates p97 [12], while Ufd1-Npl4 has no effect [55]. Understanding how cofactors modulate the activity of p97 has been challenging because cofactors are generally highly disordered proteins, which makes structural studies of the complexes challenging. Therefore, most of the structural information on p97-cofactors complexes is limited to the high-resolution structures of interacting domains and peptides [65,66,67] and low-resolution cryo-EM structures [28,57]. The Ufd1 cofactor is partially resolved in the high-resolution cryo-EM structure of the yeast and human Ufd1/Npl4-p97 complexes [39,60]. Finally, links have been reported between nucleotide occupancy, p97 conformation and cofactor binding. For example, UBXD1 preferably binds to the N-domains of p97 in its down position (when the N-domains of p97 are coplanar to the D1 ring, the conformation adopted by p97 when the D1 domains are bound to ADP; Figure 2B) [59]. The SHPN motif of p47 interacts with p97 when the N-domain is in its up position (above the D1 ring, the conformation adopted by p97 when the D1 domains are bound to ATP; Figure 3A) [47]. The N-domains of Cdc48 were in their up positions in the structures obtained with an engaged substrate and Ufd1-Npl4 [39] or Shp1, the yeast homologue of p47 [38].

Understanding the effect of cofactors on p97 function and structure remains an area of high interest, and further studies are needed. Since the function of p97 in different cellular pathways is defined by cofactors, understanding the role of cofactors will facilitate the design of drugs that target specific pathways associated with p97 in cells.

### 3.2. Effect of Disease-Causing Mutations on the Structure and Function of p97

A large number of missense mutations at more than 30 positions in the p97 sequence have been associated with neurodegenerative diseases, including inclusion body myopathy with early-onset Paget disease and frontotemporal dementia (IBMPFD, also referred to as MSP1 or VCP-associated multisystem proteinopathy), amyotrophic lateral sclerosis (ALS), and Charcot–Marie–Tooth disease. The accumulation of ubiquitinated aggregates is a common pathological feature of these diseases, correlating with the role of p97 in the protein degradation pathways [68]. Intriguingly, the majority of these disease-causing mutations are clustered at the N-D1 interface of p97 [69]. They result in increased ATPase activity but do not impair the assembly of p97 as a hexamer [70].

The structural impact of mutations located at the N-D1 interface was first analysed using crystallography and biochemical approaches with the ND1 fragment [45]. These studies suggested that the IBMPFD mutations altered the timing of the ADP-bound conformation, destabilising the co-planar conformation of the N-domain and favouring its upward movement. Truncated N-D1 fragments bearing these mutations showed higher ATPase activity, compared to the wild type, and lower affinity for ADP [71]. NMR spectroscopy quantified the effect of mutations in the ND1 interface and showed that the mutations resulted in the destabilisation of the N-domains from their co-planar conformation and resulted in their movement upwards, and this even in the presence of ADP [59,72]. This is strikingly different from reported studies conducted with the wild-type protein for which the N-domains are coplanar to D1 in the presence of ADP and move upwards in the presence of ATP (Figure 3). The NMR study showed that certain mutations had a stronger destabilising effect than others, with, for example, the upwards movement of the N-domain being inversely proportional to the charged/hydrogen bonding nature of the residues at position 155. A more complete analysis of the effect of IBMPFD mutations was obtained with the full-length protein and was conducted using single-particle cryo-EM [73]. This study confirmed the destabilising effect of some mutations in the context of the full-length protein (Table 1 and Table 2). It showed that in addition to the charge at position 155, the upwards shift of the N-domain also resulted in the ordering of the N-D1 linker into an α-helical structure between residues 190 and 199 (mutant R191Q) and in the destabilisation of the interprotomer interaction with the D1 ring (mutant A232E). This study also showed that mutations in the D1-D2 linker prevented the upward motion of the N-domain upon ATP binding (p97E470D and p97D592N).

The increased flexibility of the N-domains (that is, the destabilisation from the N-down conformation) impacts cofactor binding, favouring the binding of certain cofactors [78,79,80], associating the conformation of the N-domain with increased binding of a cofactor such as Ufd1/Npl4 [76] and impairing the binding of cofactors such as UBXD1 [59] and p47 [13]. An increase in the rate of substrate unfolding, a function dependent on Ufd1/Npl4, has been observed with IBMPFD mutants [76], which is in agreement with the accumulation of protein aggregates in IBMPFD patients [68]. Impaired caveolar endocytosis, a process dependent on the interaction of p97 with UBXD1, has also been observed in patients [81].

Different conformational changes in p97 wild-type and disease-associated mutations affect the interaction with cofactors and potential inhibitory effects, as p47 inhibited the ATPase activity of the p97 wild type in a two-phase model, with a higher inhibitory effect at a lower concentration of p47, while it had a single inhibitory phase in a p97 disease-causing mutation [13].

### 3.3. Regulating p97 Activity Using Small Molecules

**Figure 5:**
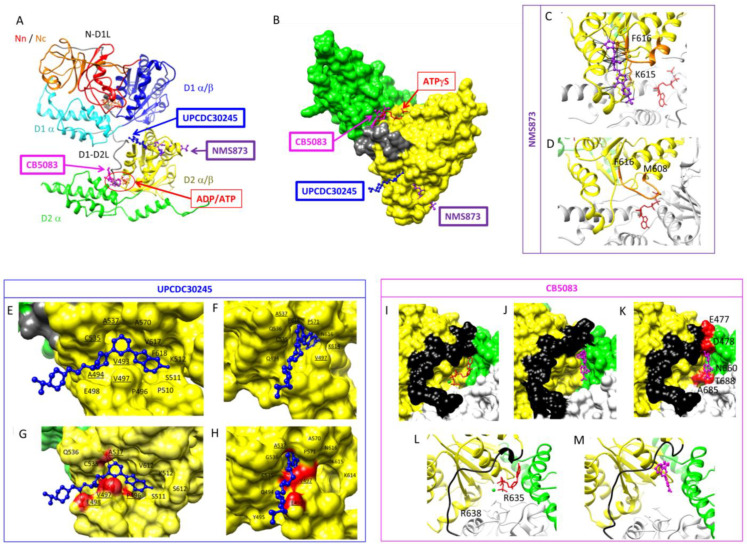
The modes of action of p97 inhibitors. (**A**,**B**) Position of inhibitors. Cartoon representation of a p97 monomer superimposed with UPCDC30245 (PDB ID: 5FTJ), CB5083 (PDB ID: 7RLI), NMS873 (PDB ID: 7LMY) and ADP/ATP (PDB ID: 5FTN) at their respective binding sites (**A**). All inhibitors interact with the D1D2 linker and the D2 domain of p97, shown as surface representations (**B**). (**C**,**D**) Mode of inhibition of NMS873. NMS873 interacts with 19 residues of its binding pocket, as indicated by black lines. Importantly NMS873 interacts with residues K^615^, N^616^ and F^618^ in the ISS motif (orange; (**C**)) and presumably prevents this motif from undergoing the conformational change observed during substrate unfolding (PDB ID: 7LN4; (**D**)). (**E**–**H**) Mode of inhibition of UPCDC30425. Two orthogonal views (**E**,**F**) of UPCDC30245 in its binding pocket in the D2 domain (PDB ID: 5FTJ). Residues lining the pocket are indicated, and potentially interacting residues are underlined. Residues 571, 614, 615 and 616 that cover the binding pocket have been removed for clarity in panels (**E**,**G**). Steric clashes between UPCDC30245 and residues of p97 in the ATPγS bound structure (PDB ID: 5FTN; (**G**,**H**), clashing residues are coloured red). Binding of UPCDC30245 presumably prevents the conformational change occurring upon ATP binding. (**I**–**M**) Mode of inhibition of CB5083. CB5083 (purple; (**J**)) binds to the nucleotide binding site (ATPγS, red; (**I**)). Binding of CB5083 presumably prevents the D1D2 linker from adopting its ATP-bound conformation, as seen by clashes between CB5083 and residues E477, D478, N660, A685 and T688 (red; (**K**)). Moreover, the Arginine fingers R635 and R638 that interact with the gamma phosphate to stabilise the ATP conformation (**L**) do not interact with CB5083 (**M**).

Since p97 has been identified as a target for treating neurodegenerative diseases [82,83], cancer [84] and viral infection [11], a number of p97 inhibitors have been identified, including ATP competitive and allosteric inhibitors [10,14]. Several reviews have described the discovery of the different classes of small-molecule inhibitors [84,85]. The search for novel and more specific inhibitors is ongoing. Several p97-dependent functions are affected by inhibitors, including autophagy, cancer cell growth, endoplasmic reticulum-associated degradation and ubiquitin fusion degradation [86,87,88]. Several structural studies have determined the structures of p97 in complexes with inhibitors and have revealed the mechanisms by which these small molecules inhibit the function of p97 [31,40,73,89]. Structures of p97 in complex with three different inhibitors occupying three different sites in p97 have been determined (Figure 5A,B). In this section, we will summarise the current knowledge about the mode of action of p97 inhibitors.

#### 3.3.1. ATP-Competitive Inhibitors

Designing ATP-competitive inhibitors presents challenges with respect to specificity, as they may also target several other cellular ATPases, and their use in the clinic may be associated with a broad array of unwanted side effects. The ATP competitive inhibitor DBeQ targets both ATPase domains [88,90] and is a reversible inhibitor of p97 with an IC50 of 2.6 µM. ML240 and ML241 are derivatives of DBeQ and D2 selective inhibitors with IC50s of 0.11 µM [91,92]. Both ML240 and ML241 inhibit the degradation of p97 substrates in a proteasome-dependent manner, impair ERAD and arrest cancer cell growth [91]. NMS-859 is a covalent inhibitor (IC50: 0.37 µM) that covalently modifies Cys522 in the D2 domain and prevents the ATPase activity of p97 [93]. CB-5083 [86] is a selective, potent and orally bioavailable inhibitor of p97 (IC50 11 nM) with a demonstrated inhibitory effect in solid tumour and hematological xenograft models [94]. The crystal structure of the p97:CB-5083 complex revealed differences in size and shape between the D1 and D2 ATP binding sites that explained specificity [89]. Binding of CB-5083 prevents the change of conformation of the D1D2 linker, as clashes would occur between CB-5083 and residues E477, D478, N660, A685 and T688 (Figure 5I–M). Despite promising properties, the CB-5083 phase I clinical trial failed due to an off-target effect on visual function due to the inhibition of phosphodiesterase 6 [95]. A second-generation inhibitor, CB-5339, less active on phosphodiesterase 6, was developed [96] and is currently in a phase I trial for treating hematological malignancies and solid tumours in China and the USA [97]. The development of new-generation inhibitors based on knowledge of the properties of inhibitors and their interactions with proteins is ongoing.

#### 3.3.2. Allosteric Inhibitors

Allosteric inhibitors offer better promise for specificity [98]. Eeyarestatin l (Eer1) is one of the allosteric inhibitors targeting p97, affecting the ERAD pathway [99] and inducing cell death in hematological cancer cells [100]. SPR studies and trypsin digestion analysis suggested that Eer1 interacted with the D1 domain of p97 with an estimated Kd of 5–10 µM [87]. Still, the exact binding site and the mode of action of EerI remain unknown. NMS-873 was identified in a high-throughput screening for chemical compounds impairing p97 ATPase activity. This compound activates the unfolded protein response, interferes with autophagy and induces cancer cell death [93]. It has an IC50 of 30 nM [93]. An allosteric inhibitor, UPCDC30245, was optimised through a medicinal chemistry approach after the discovery of a 2-phenyl indole scaffold through high-throughput screening [101]. The structure of UPCDC30245 in complex with p97 was determined at high resolution using single-particle cryo-EM [31]. With an IC50 of 27 nM, UPCDC30245 was shown to bind in a tunnel lined with residues 483–498 and 523–534, with the fluorinated indole of the inhibitor pointing toward the centre of p97 (Figure 5E–H). The structure of p97: UPCDC30245 was solved with ADP in D1 and D2. The conformation of p97 solved with ATPγS in D2 is incompatible with the binding of UPCDC30245 due to steric clash with P^496^, V^497^, E^498^, F^496^ and A^537^. This observation indicates that UPCDC30245 prevents ATP binding to D2 and/or decouples ATP binding and the associated conformational change.

**Figure 6:**
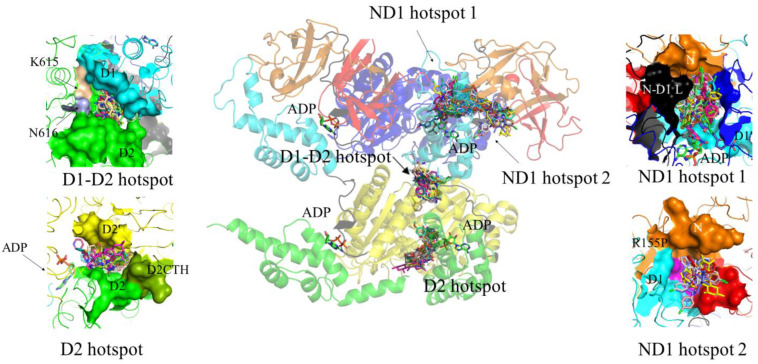
Plausible allosteric inhibitor-binding hotspots of p97. All the molecular docking simulations were carried out using the Lamarckian genetic algorithm used by Autodock Vina part of the MGLTools [105]. The receptor for the docking was prepared from two adjacent monomers of high-resolution hexameric p97 structure (PDB ID: 5FTK). MGL tools were used to add polar hydrogens and Kollman charges to the receptor structure. The coordinate files of the inhibitor ligands were prepared and energy-optimised using JSME molecular editor [106] and prodrg [107], respectively. Finally, blind docking was performed using autodock vina with a grid box size of 50 Å × 50 Å × 54 Å, 1.0 Å grid spacing and exhaustiveness of 32. The interaction profiles of each tested ligand with the receptor were analysed by PyMol (Schrödinger). The middle panel shows blind docking of four plausible allosteric inhibitor-binding pockets of p97 dimer (5FTK). The upper left panel shows the D1-D2 inhibitor-binding hotspot. The pocket comprises amino acid residues from the D1 domain, D1-D2 linker and D2 domain from the neighbouring monomer (D2′). Two previously identified amino acid residues, K^615^ and N^616^, important for binding NMS873 and MSC1094308, are colour-coded as khaki and purple, respectively. The lower left panel shows the D2 inhibitor binding hotspot. This pocket comprises D2 domains of two adjacent monomers. The C-terminal helix residues (D2CTH) from one monomer are colour-coded as light green. The arrow indicates the bound ADP molecule close to this hotspot. The upper right panel shows ND1 inhibitor-binding hotspot 1. This hotspot is surrounded by amino acid residues from the N-domain, N-D1 linker (N-D1L) and D1 domain from one monomer, as well as the amino acid residues from the D1 domain from the neighbouring monomer (D1′). The bound ADP molecule close to this hotspot is indicated by the arrow. The lower right panel shows ND1 inhibitor-binding hotspot 2. This hotspot is composed of amino acid residues from the N- and D1 domains from the same monomer. This inhibitor-binding hotspot also homes one of the predominant diseases related to mutational hotspot residues of p97 (R155, colour-coded as magenta).

Azido derivatives combined with UV cross-linking and mass spectrometry analysis identified the binding site of NMS-873 to be in loops at the interface between the D2 domains of two adjacent protomers [93]. The proposed mode of action of NMS-873 is to interfere with the intersubunit signaling (ISS) motif, a motif important in the communication between the D2 nucleotide binding site of two adjacent protomers. According to the model, the interaction of the inhibitor with the ISS motif would impede the movement of the arginine finger, preventing the conformational change of ATP hydrolysis from being propagated to the adjacent monomer and consequently freezing D2 in the ADP-bound state [93]. Recently, the mode of action of NMS-873 was also uncovered using cryo-EM (Figure 5C,D). This study demonstrated that the inhibitor interacts with the ISS motif and prevents subsequent conformational changes during substrate translocation [40]. NMS873 interacts with 19 residues of its binding pocket, as indicated by black lines. Importantly NMS873 interacts with residues K^615^, N^616^ and F^618^ in the D1D2 linker (Figure 5C) and prevents the ISS motif from undergoing the conformational change observed during substrate unfolding (PDB ID: 7LN4; Figure 6D).

MSC1094308 (IC50 7.2 μM) has been proposed to act via a similar molecular mechanism based on mutagenesis analysis of a key residue in NMS-873 interaction [102]. A screen for ND1 binders identified NW1028 and NW1030 with a KD of 285 and 7.3 nM, respectively, with NW1028 inhibiting the ATPase activity of D1 and NW1030 increasing it. A mass-spectrometry analysis combined with docking experiments pointed to a binding site at the interface between N and D1 close to the ND1 linker. Interestingly, these compounds affected only a subset, yet a different subset, of p97 functions [103]. Xanthohumol binds to the N-domain of p97 and inhibits autophagosome maturation [104]. The exact binding site and mode of action of Xanthohumol remain unknown.

#### 3.3.3. Potential Additional Binding Sites for Allosteric Inhibitors

To date, three binding sites for p97 inhibitors have been described at the structural level. A previous targeted docking experiment predicted two main inhibitor-binding sites [85]. One site is the nucleotide binding site in D2, the binding site for CB5083 [89] and the predicted binding sites for LC-1028, NMS-529, PPA and curvularin [85] and FQ-393 [74]. The other site is at the surface of D2 and is the binding site for UPCDC30245 [31] and NMS873 [40]. To identify potential additional binding sites and inhibitor binding modes, we performed blind docking of inhibitors, as reported in recent reviews [10,14]. Blind docking identified four main sites or hot spots where allosteric inhibitors could bind (Figure 6). The first hotspot comprises amino acid residues from the D1 domain, the D1-D2 linker and the D2 domain from the neighbouring monomer (D2′) (D1-D2 hotspot; Figure 6). This site is lined by the amino acids K^615^ and N^616^, which have previously been found to be important for the binding of NMS873 [93] and MSC1094308 [102]. The second hotspot is located in D2 at the interface between two adjacent monomers to close α9 (Asn750-Gln760) of one protomer (D2 hotspot; Figure 6). It is close but distinct from the nucleotide binding site in D2. The third hotspot (ND1, hotspot 1; Figure 6) is surrounded by amino acid residues from the N-domain, the N-D1 linker (N-D1L) and the D1 domain from one monomer as well as the amino acid residues from D1 domain from the neighbouring monomer (D1′). No inhibitor has yet been reported or predicted to bind here, although LC-MS/MS of p97 incubated with LC-1028 identified Cys105 (located in ND1 hotspot 1) as an off-target labelling site [75]. The fourth hotspot (ND1 hotspot 2; Figure 6) is also located in the ND1 region. It is composed of amino acid residues from the N- and D1 domain from the same monomer and homes one of the predominant disease-related mutational hotspot residues of p97 (R155). The D1 and D2 nucleotide binding sites were not identified as binding sites in our blind docking experiment because the ADP nucleotides in D1 and D2 were not removed.

**Figure 7:**
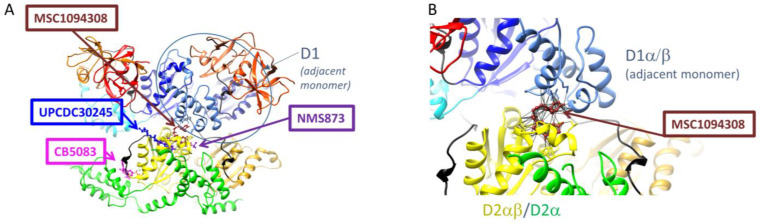
A Possible mode of inhibition of MSC1094308. The docking methodology is the same as that described in Figure 6. Docking pause of MSC1094308 to p97 (5FTK, ADP/D1-ADP/D2). MSC1094308 is located roughly in the same hotspot as inhibitors CB5083 and UPCD30245 (**A**) but is predicted to interact both with residues in the D2αβ sub-domain and a residue in the adjacent D1α/β domain (**B**).

Notably, one of the identified hotspots (at the D1-D2 interface) was found to be the most preferred binding site of MSC1094308 (Figure 7), which is consistent with a photo affinity-based crosslinking study that identified K^615^ and N^616^ as key residues for the interaction [102]. Targeted docking was conducted at this site with different conformational states of p97 (PDB IDs: 1R7R, 5FTM, 5FTN and 7LN5). Inhibitor MSC1094308 was found to bind only to two different conformational states of p97 (5FTK and 5FTN) at the D1-D2 interface. In these conformations, D1 and D2 are both occupied with ADP, or both occupied with ATPγS. The docking solution showed that MSC1094308 interacts with residues in the D2α/β sub-domain as well as residues in the D1α/β sub-domain. This mode of interaction suggests that MSC1094308 may inhibit p97 in a different manner to NMS873 and UPCDC30245, namely, by preventing sliding and rotation of these two sub-domains that occur upon ADP/ATP exchange in D2 and during substrate unfolding.

## 4. Discussion and Future Perspectives

In recent years, p97 has emerged as a central component of protein homeostasis [25]. It functions upstream of the 26S proteasome and prepares well-folded protein substrates for degradation through the proteasome. Mutations in p97 are associated with neurodegenerative diseases known as inclusion body myopathy with Paget disease and frontotemporal dementia (IBMPFD). The apparent symptom of the disease is the presence of protein aggregates in muscle and brain tissues of IBMPFD patients, which implicate the changes in protein homeostasis through p97 [83,108]. Elevated p97 expression has also been reported in several types of cancers, including breast, lung, pancreatic and colorectal cancer. Due to their uncontrolled cell division, cancer cells manifest high protein turnover rates and are highly dependent on protein homeostasis through the proteasome system. Likewise, proteasome inhibitors have been proven to be potential drug candidates for treating patients with multiple myeloma [109].

However, the growing concern of cancer drug resistance and the inefficiency of proteasome inhibitors against solid tumours demands a new antineoplastic drug target. In this context, p97 can serve as a promising drug target owing to its central role in eukaryotic protein homeostasis. Moreover, disruption of p97 function results in the death of cancer cells, which correlates with its essential role in the cellular metabolism of cancer cells [110,111,112]. As such, p97 has attracted much attention from pharmaceutical companies as a potential target in cancer therapy [14,94,113,114]. Several p97-dependent functions are affected by inhibitors, including autophagy, cancer cell growth, endoplasmic reticulum-associated degradation and ubiquitin fusion degradation.

The success and limitations in developing drugs that target the ubiquitin-proteasome system, such as Bortezomib and Carfilzomib [115,116,117,118], encouraged research into a mechanochemical machine, p97, as a target for cancer therapy. Several different inhibitors have been developed for p97 which target the ATPase activity of the protein through competitive inhibition or act allosterically. However, most of these inhibitors lack specificity toward p97 and exhibit off-target effects when administered in vivo.

Despite the considerable interest of pharmaceutical companies in targeting p97, no drug targeting p97 has yet made it to the market. Since p97 recruits its substrates in a vast number of cellular pathways with the help of a large number of cofactors (>20), this offers opportunities for designing pathway-specific inhibitors. Two distinct pieces of experimental evidence highlight the potential for more specific inhibitors: firstly, disease-associated mutants (such as R95G) show an increased IC50 for certain inhibitors (e.g., ML240); and, secondly, certain cofactors (e.g., cofactor p47) decrease the inhibition potential of small molecules, such as ML240, ML241 and CB5083, while others (e.g., Ufd1-NPL4) do not affect CB-5083 inhibition efficiency [85,109,110].

Here, we have reviewed recent works that have advanced our understanding of the molecular mechanisms by which p97 functions as a molecular unfoldase. We have also described some mechanisms known to regulate its activity. A better understanding of these regulatory mechanisms is essential for designing more specific therapeutic drugs targeting p97 for the treatment of cancer, neurological diseases and potentially infectious diseases, including parasitic diseases [9]. It is worth noting that our current understanding of the functions of p97 is based on bulk studies and average structures. However, the ATPase p97 and its cofactors are by and large flexible molecules. The N-domains of p97 move up and down around the D1 ring and are poorly defined in cryo-EM structures. p97′s cofactors have the biophysical characteristics of fuzzy proteins, with small ordered domains separated by flexible regions. A better understanding of the regulation of p97 activity will invariably require single-molecule studies and hybrid structural biology approaches that characterise the dynamic properties of the complexes and their modifications in cellular contexts and conditions.

## 5. Conclusions

The AAA+ ATPase p97 is an abundant protein in the cell that performs a variety of functions. This multifunctional protein is guided to different pathways in the cell via interaction with cofactors or adaptors. p97 uses ATP as a fuel and undergoes conformational changes to process substrates. Mutation in p97 causes neurodegenerative diseases, and the inhibition of p97 has been identified as a treatment for cancer and infectious diseases. This review has presented the current detailed understanding of the mechanochemical activity of p97 during the ATPase cycle and upon substrate binding, as well as the molecular mechanism by which small molecules modulate the function of p97. Using docking, we have proposed a novel mechanism for p97 inhibition by MSC1094308 and identified three additional potential binding sites for small molecules. This review highlights the potential for the discovery of new p97 inhibitors.

## Figures and Tables

**Table 1:** List of full-length (FL) and ND1 structures of the wild-type p97 hexamers determined in the absence of substrates and cofactors.

Construct	PDB/ EMD ID	Method	Resolution (Å)	Ligand D1/D2	Position of N	Ref. Year
FL	3CF1	X-ray	4.40	ADP/ADP-ALFx	Down	[30,32] 2003-08
FL	3CF3	X-ray	4.25	ADP/ADP	Down	[30,32] 2003-08
FL	3CF2	X-ray	3.50	ADP/ AMP-PNP	Down	[30,32] 2003-08
FL	1R7R	X-ray	3.60	ADP/-	Down	[74] 2003
FL (Δ709–728)	5C18	X-ray	3.30	ATPγS/ATPγS	Down	[33] 2016
FL (α9-D4) *	5C19	X-ray	4.20	-/-	Down	[33] 2016
FL (α9-D4) *	5C1A	X-ray	3.80	ATPγS/ATPγS	Down	[33] 2016
FL	5FTK/3296	EM	2.4	ADP/ADP	Down	[31] 2016
FL	5FTN/3299	EM	3.3	ATPγS/ATPγS	Up	[31] 2016
FL	5FTL/3297	EM	3.3	ADP/ADP	Down	[31] 2016
FL	5FTM/3298	EM	3.2	ADP/ATPγS	Down	[31] 2016
N-D1	1E32	X-ray	2.90	ADP	Down	[75] 2001
N-D1	5DYI	X-ray	3.71	ADP	Down	[71] 2015
FL-Npl4/Ufd1	21824, 21825, 21826	EM	3.8 3.7 3.9			[60] 2021
FL	7JY5/22521	EM	2.89	ATPγS/ATPγS		[60] 2021
FL-Npl4/ Ufd1-Ub-Eos	21827, 21828, 21829, 21830	EM	4.2 4.3 4.5 3.5			[60] 2021
FL-Npl4/Ufd1-Ub6-FOM, 3 classes	7LMZ/23443 7LN0/23444 7LN1/23445	EM	3.06 2.98 3.40	ATP/ATP ATP/ATP ATP/ATP	Up Up Up	[40] 2021
FL-Npl4/Ufd1-Ubn-Eos-FOM, 3 classes	7LN2/23446 7LN3/23447 7LN4/23448	EM	3.63 3.45 3.00	ATP/ATP ATP/ATP ATP/ATP	Up Up Up	[40] 2021
FL-Npl4/Ufd1-Ubn-Eos-CHAPSO, 2 classes	7LN5/23449 7LN6/23450 23451	EM	3.09 3.58 3.77	ATP/ATP ATP/ATP	Up Up	[40] 2021
FL-Npl4/Ufd1-Ub6-NMS-873-FOM	7LMY/23442	EM	2.40	ATP/ATP	Up	[40] 2021
FL-Npl4/Ufd1-Ub6-NMS-873-CHAPSO	23452 23453 23454 23455 23456 23457 23458	EM	2.87 3.69 6.00 3.58 4.25 3.47 3.65			[40] 2021
Dodecamer FL/FL	7VCS/31894	EM	3.32	ATPγS/ATPγS ATPγS/ATPγS	Up/up	[49] 2022
Dodecamer FL/FL	7VCU/31896	EM	3.15	ATPγS/ADP ATPγS/ADP	Down/ down	[49] 2022
FL	7VCT/31895	EM	3.21	ATPγS/ADP	Down	[49] 2022
FL	7VCV/31897	EM	3.21	ATPγS/ATPγS	Up	[49] 2022
FL	7VCX/31899	EM	3.24	ATPγS/ATPγS	Down	[49] 2022

* α9-D4 is a quadruple aspartate mutant (N750D/R753D/M757D/Q760D) designed to prevent dodecamer formation mediated by the C-terminal helix (α9).

**Table 2:** Structures of disease-associated p97 mutants (only including high-resolution structures with deposited atomic models).

Construct	PDB/ EMD ID	Method	Resolution (Å)	Mutation	Ligand D1/D2	Position of N	Ref. Year
FL/UN	20730	EM	4.26	A232E	?	Up	[76] 2019
FL	-	EM	3.7	R95G	?	Mixture	[77] 2019
N-D1	4KO8	X-ray	1.98	R155H	ATPγS(D1)	Up	[71] 2013
N-D1	4KOD	X-ray	2.96	R155H	ADP(D1)	Down	[71] 2013
N-D1	4KLN	X-ray	2.62	A232E	ATPγS(D1)	Up	[71] 2013
N-D1	5DYG	X-ray	2.20	L198W	ADP (D1)	Down	[44] 2015
N-D1	3HU1	X-ray	2.81	R95G	ATPγS(D1)	Up	[45] 2010
N-D1	3HU1	X-ray	2.85	R86A	ATPγS(D1)	Up	[45] 2010
N-D1	3HU3	X-ray	2.20	R155H	ATPγS(D1)	Up	[45] 2010
FL	7RL6/24518	EM	3.7	R155H	ADP (D1) ADP (D2)	Down	[73] 2021
FL	7RL7/24519	EM	3.0	R155H	ATPγS (D1) ATPγS (D2)	Up	[73] 2021
FL	7RL9/24522	EM	3.3	R191Q	ADP (D1) ADP (D2)	Up	[73] 2021
FL	7RLA/24523	EM	3.1	R191Q	ATPγS (D1) ATPγS (D2)	Up	[73] 2021
FL	7RLB/24524	EM	3.3	A232E	ADP (D1) ADP (D2)	Down	[73] 2021
FL	7RLC/24525	EM	3.2	A232E	ATPγS (D1) ATPγS (D2)	Up	[73] 2021
FL	7RLD/24526	EM	3.4	E470D	ADP (D1) ADP (D2)	Down	[73] 2021
FL	7RLF/24528	EM	3.1	E470D	ATPγS (D1) ATPγS (D2)	Down	[73] 2021
FL	7RLG/24529	EM	3.7	D592N	ADP (D1) ADP (D2)	Down	[73] 2021
FL	7RLH/24530	EM	3.7	D592N	ATPγS (D1) ATPγS (D2	Down	[73] 2021

## Data Availability

Not applicable.
